# Enhancing ontology-driven diagnostic reasoning with a symptom-dependency-aware Naïve Bayes classifier

**DOI:** 10.1186/s12859-019-2924-0

**Published:** 2019-06-13

**Authors:** Ying Shen, Yaliang Li, Hai-Tao Zheng, Buzhou Tang, Min Yang

**Affiliations:** 1 School of Electronics and Computer Engineering, Peking University Shenzhen Graduate School, Shenzhen, 518055 People’s Republic of China; 2 Alibaba Group, Bellevue, WA USA; 3 School of Information Science and Technology, Graduate School at Shenzhen, Tsinghua University, Shenzhen, 518055 People’s Republic of China; 4 Harbin Institute of Technology (Shenzhen), Shenzhen, 518055 People’s Republic of China; 5 SIAT, Chinese Academy of Sciences, Shenzhen, 518055 People’s Republic of China

**Keywords:** Ontology, Probability, Uncertainty reasoning, naïve Bayes classifier

## Abstract

**Background:**

Ontology has attracted substantial attention from both academia and industry. Handling uncertainty reasoning is important in researching ontology. For example, when a patient is suffering from cirrhosis, the appearance of abdominal vein varices is four times more likely than the presence of bitter taste. Such medical knowledge is crucial for decision-making in various medical applications but is missing from existing medical ontologies. In this paper, we aim to discover medical knowledge probabilities from electronic medical record (EMR) texts to enrich ontologies. First, we build an ontology by identifying meaningful entity mentions from EMRs. Then, we propose a symptom-dependency-aware naïve Bayes classifier (SDNB) that is based on the assumption that there is a level of dependency among symptoms. To ensure the accuracy of the diagnostic classification, we incorporate the probability of a disease into the ontology via innovative approaches.

**Results:**

We conduct a series of experiments to evaluate whether the proposed method can discover meaningful and accurate probabilities for medical knowledge. Based on over 30,000 deidentified medical records, we explore 336 abdominal diseases and 81 related symptoms. Among these 336 gastrointestinal diseases, the probabilities of 31 diseases are obtained via our method. These 31 probabilities of diseases and 189 conditional probabilities between diseases and the symptoms are added into the generated ontology.

**Conclusion:**

In this paper, we propose a medical knowledge probability discovery method that is based on the analysis and extraction of EMR text data for enriching a medical ontology with probability information. The experimental results demonstrate that the proposed method can effectively identify accurate medical knowledge probability information from EMR data. In addition, the proposed method can efficiently and accurately calculate the probability of a patient suffering from a specified disease, thereby demonstrating the advantage of combining an ontology and a symptom-dependency-aware naïve Bayes classifier.

## Background

An ontology is a set of concepts in a domain space, along with their properties and the relationships between them [1]. The past couple of decades have witnessed many successful real-world applications of ontologies in the medical and health domain, such as in medical diagnosis [2], disease classification [3], clinical inference learning [4], and medical knowledge representation and storage [5].

Despite their effectiveness of previous studies, existing ontologies for the medical domain are missing an important component: the knowledge-triplet probability. Due to the uncertainty and complexity of knowledge in the medical domain, the probability of a knowledge triplet depends on its head entity and tail entity. For example, the probability of knowledge triplet (poor appetite, symptom-disease, cirrhosis) is 0.20; hence, when suffering from cirrhosis, 20% of patients have poor appetite. Such probabilities in medical knowledge are crucial for decision-making in various medical applications. Therefore, it is important to supplement medical ontologies with probability information.

An electronic medical record (EMR) is a structured collection of patient health information and medical knowledge that contains valuable information about probabilities. Thus, it can be a high-quality resource for the discovery of medical knowledge probabilities. After investigating the uncertainty regarding the actual situation of the patient, it is necessary to separate the symptoms and diseases that are possible from those that are impossible to determine which measures might be effective [6].

To overcome the challenges that are discussed above, we propose a novel knowledge acquisition method for medical probability discovery. Patients’ medical records are used to construct an ontology and train a symptom-dependency-aware naïve Bayes classifier (SDNB classifier) to evaluate the probability of a disease before we observe any symptoms and the posterior probability considering the correlations among symptoms.

To evaluate the performance of the proposed method, we conduct experiments to evaluate the combined performance of the generated ontology and the symptom-dependency-aware naïve Bayes classifier on the medical diagnostic classification task. The experimental results demonstrate that our method can effectively discover medical knowledge probabilities and accurately classify diseases and pathologies.

In addition, we evaluate the performance of the proposed method under various scenarios in disease reasoning tasks by visualizing how ontological analysis is combined with a symptom-dependency-aware weighted naïve Bayes classifier to conduct the probability estimation and how probability enhances the interactions between the user and the computer in gastroenterology disease reasoning.

Our main contributions are threefold: 1) We enrich medical knowledge graphs with probability information by discovering the knowledge-triplet probability information from EMR data, which renders the corresponding medical ontology more accurate and more applicable to medical tasks. 2) We present a method for improving the naïve Bayes classifier based on the relevance of various attributes to disease diagnosis. 3) We demonstrate that the proposed method can reliably discover knowledge-triplet probabilities for medical ontologies. We also demonstrate the viability of training naïve Bayes classifiers to support medical decision-making.

### Related work

#### Knowledge discovery from EMRs

EMR data on the phenotypes and treatments of patients are an underused data source that has much higher research potential than is currently realized. With their high-quality medical data, EMRs open new possibilities for data-driven knowledge discovery towards medical decision support. The mining of EMRs may establish new patient-stratification principles and reveal unknown disease correlations [7].

There are various medical knowledge discovery applications that are based on EMRs, including the discovery over-structured data (e.g., demographics, diagnoses, medications, and laboratory measurements) [8] and unstructured clinical text (e.g., radiology reports [9] and discharge summaries [10]). The research can be divided into entity discovery [11], phenotype extraction [12], disease topic discovery [13], temporal pattern mining [14], and medical event detection [15]. Several NLP techniques have been developed for clinical texts, e.g., coreference resolution [16], word sense disambiguation [17] and temporal relations [18]. Many studies have attempted to create annotated corpora [19] to facilitate the development and testing of these algorithms, which has also been the emphasis of the biomedical and clinical informatics community.

#### Probability discovery

In the literature, ontologies have been extensively studied with naïve Bayes classifiers via various approaches, such as document classification [20], ontology mapping [21, 22], and sentiment analysis [23]. However, the combined application of an ontology and a naïve Bayes classifier in medical uncertainty reasoning remains relatively new territory that is underexplored.

A naïve Bayes classifier is a probabilistic classifier that is based on Bayes’ theorem that imposes strong (naive) independence assumptions between the features [24]. For example, the disease diagnosis module for the Global Infectious Disease and Epidemiology Network (GIDEON) [25] was developed using a naïve Bayes classifier that evaluates disease probabilities based on the patient’s background, incubation period, symptoms and signs, and laboratory test results. Naïve Bayes classifiers have also been applied in many clinical decision support tasks, e.g., curing mammographic mass lesions [26], optimizing brain tumor treatment [27], and predicting the likelihood of a diabetic patient getting heart disease [28].

However, such fruitful results are subject to the assumption that attributes (symptoms) are independent from each other conditioned on the class variable (disease) [29]. This assumption of attribute independence need not necessarily hold true in disease diagnostic reasoning because a symptom can be strongly correlated with many diseases or symptoms [30]. For example, the symptom “diarrhea” may cause serum-electrolyte-disturbance–associated symptoms, e.g., hypokalemia and hyponatremia, while “hypokalemia” can cause decreased intestinal peristalsis, thereby leading to loss of appetite, nausea, and constipation. Therefore, the assumption of attribute independence of naïve Bayes classifiers may severely reduce its diagnostic accuracy.

#### Ontology enrichment

Many studies have constructed ontologies, including Freebase, DBpedia, and Disease Ontology (DO) [31]. These ontologies often suffer from incompleteness and sparseness since most of them have been built either collaboratively or semiautomatically. Thus, it is necessary to supplement these ontologies with extra information. An ontology can be enriched via two approaches: The first is to enrich the distributed knowledge representation by incorporating extra knowledge into knowledge embeddings [32]. The other is to reconstruct the ontology with new elements, such as probability information [33], temporal information [34], and space constraints [35]. In this study, we exploit the probability information in the ontology, which has received little attention so far.

#### Symptom-disease network reasoning

In the medical field, many studies explore the elucidation of the relationship between the molecular origins of diseases and their resulting symptoms. For example, Hidalgo et al. [36] introduce a new phenotypic database that summarizing correlations that were obtained from the disease histories of more than 30 million patients in a phenotypic disease network. Zhou et al. [37] use large-scale medical bibliographic records and the related medical subject heading (MeSH) metadata from PubMed to generate a symptom-based network of human diseases, where the link weight between two diseases quantifies the similarity of their corresponding symptoms. The main difference between our work and these existing works is that we incorporate AdaBoost optimization with a medical-specific OR value evaluation that can identify the variables of health features and attributes to evaluate the co-occurrence frequency among symptoms in the EMRs. In addition, the final output of our task is an ontology rather than a symptom-based network. The annotations in the generated ontology, such as the disease introduction, disease/syndrome synonym, category, pathology, department, part of body, and lesion, can provide disease-related details to the user and facilitate clinical decision-making.

## Results

### Ontology component analysis

First, we evaluate the quality of the generated ontology, which is the final output of our task. Based on over 30,000 deidentified medical records, we explore 336 gastrointestinal diseases and 81 related symptoms. Among these 336 gastrointestinal diseases, the probabilities of 31 diseases are obtained via our method. These 31 probabilities of diseases and 189 conditional probabilities between diseases and symptoms are added to the generated ontology. We cannot obtain the probabilities of other diseases since they are difficult to subjectively quantify or their statistical results are unconvincing due to insufficient medical records (e.g., there are only 2 medical records that correspond to gastrointestinal stromal tumors).

A subset of the diseases and their syndromes, along with their conditional probabilities, are summarized in Table 1.

**Table 1:** Examples of the diseases and their syndromes and conditional probabilities

Disease	Syndrome and Conditional Probability
Acute pyelonephritis	(fever, 0.2), (shaking, 0.1), (frequent urination, 0.1), (urinary incontinence, 0.1), (odynuria, 0.1), (stomachache, 0.1), (urine turbidity and urinary smell, 0.1), (nausea, 0.05), (vomiting, 0.05), (headache, 0.05), and (sore all over, 0.05)
Acute interstitial nephritis	(oliguria, 0.6), (fever, 0.1), (rash, 0.1), and (joint pain, 0.1)
Chronic interstitial nephritis	(night time urination, 0.1), (foam in urine, 0.5), (blaze, 0.2), and (white nails, 0.2)

**Fig. 1:**
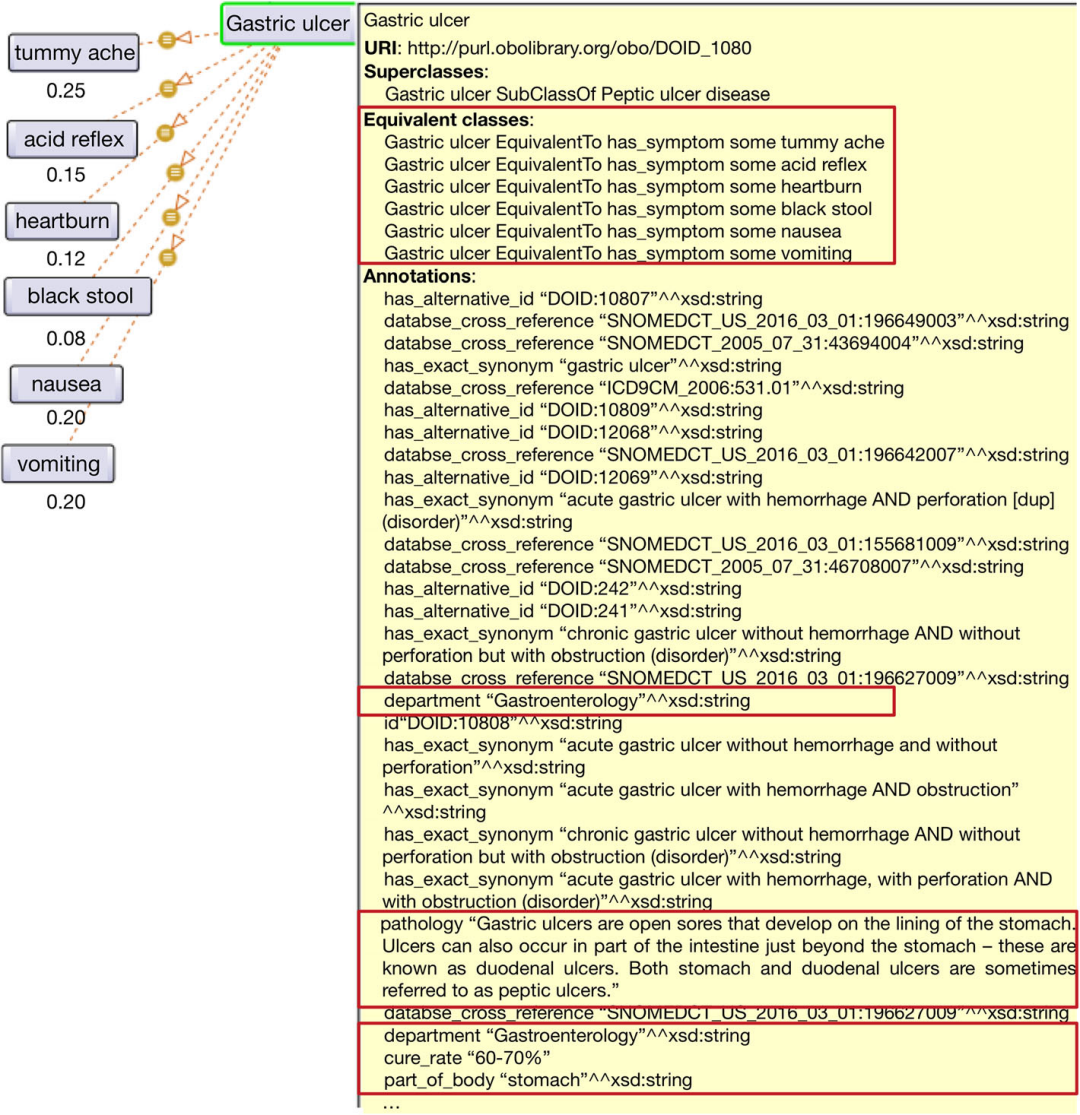
Ontology class: Gastric ulcer

Figure 1 is a subgraph of the generated ontology. For the disease “gastric ulcer”, the solid lines represent the taxonomy of the class relationships, while the dotted lines indicate the relationships between diseases and their relevant symptoms. The numbers on the dotted lines represent the occurrence probabilities of the symptoms and the corresponding diseases. We observe the following:

1. Disease-symptom mentions are identified via the proposed method. For example, the triplet (acid reflex, symptom-disease, gastric ulcer) indicates that acid reflex is a symptom of a gastric ulcer, which is useful for analyzing possible clinical signs and predicting possible subsequent probabilities of diseases.
2. The discovery of disease-relevant relationships, including disease-lesion, disease-pathology, disease-susceptible population, disease-part of body, and disease-cure rate, is also helpful for gaining insight into the proposed method.
3. The included probabilities can contribute to gastroenterology diagnosis for medical applications. The probabilities of knowledge triplets (nausea, symptom-disease, gastric ulcer) and (tummy ache, symptom-disease, gastric ulcer) are 0.20 and 0.25, respectively; hence, if suffering from a gastric ulcer, the occurrence probability of nausea is nearly the same as that of tummy ache.

### Diagnostic classification

To evaluate the performance of the knowledge-triplet probability of the proposed method, we conduct experiments on the diagnostic classification task, namely, the classification of a disease or pathology.

As a test set, 1660 medical records were randomly selected and analyzed to identify the presence or absence of cirrhosis. In our pre-experiment, we adopted the 6-fold cross-validation method. The results of each cross-validation experiment were highly similar because the medical record text that we used was homogeneous and of high quality. Therefore, we randomly selected 1660 records as the test set in the current study.

In the medical record, the most important disease from which the patient suffers is listed first and the complications are listed subsequently. This study only focused on the first disease that is listed in the medical record. Based on the doctors’ diagnosed cases, we calculate and compare the classification accuracy of the generated ontology (SDNB ontology) in four scenarios: (a) without the naïve Bayes classifier (SDNB ontology); (b) with the original naïve Bayes classifier (SDNB ontology + NB); and (c) with an improved naïve Bayes classifier that is based on the co-occurrence frequency, which was presented in [38] (SDNB ontology + improved NB); and (d) with a symptom-dependency-aware weighted naïve Bayes classifier that is realized via odds ratio (OR) value [39] evaluation and AdaBoost optimization (SDNB ontology+ SDNB classifier).

For the first scenario, we use the original ontology without the newly added probabilities and apply the path ranking algorithm (PRA) [40] to model the ontology relationships and train the classifier for each relationship. In the ontology, a relationship path can be formed by connected ontology triplets. For example, (disease, alias, disease) and (disease, corresponding symptoms, symptoms) can be connected as a path. Considering the ontology as a directed graph, PRA adopts the relationship path as a feature and represents all the relationship paths in the ontology as feature vectors. Afterwards, the classifiers are trained to identify the relationships between the entity pairs.

For the third scenario, we designed an improved Naïve Bayes classifier that is based on syndrome correlations. The correlation between symptoms S_ij1_ and S_ij2_ can be calculated via Equation (1), where P((S_ij1,_S_ij2_)| D_f_) denotes the class conditional probability of (S_ij1,_S_ij2_) and P(S_ij1_| D_f_) and P(S_ij2_| D_f_) denote the class conditional probabilities of S_ij1_ and S_ij2_, respectively. If P((S_ij1,_S_ij2_)| D_f_) > P(S_ij1_| D_f_) ∙ P(S_ij2_| D_f_) , S_ij1_ and S_ij2_ are considered positively correlated; otherwise, they are negatively correlated. If ${\mathrm{Corr}}_{\left({\mathrm{S}}_{\mathrm{ij}1,}{\mathrm{S}}_{\mathrm{ij}2}\right)\left|{\mathrm{D}}_{\mathrm{f}}\right.}=1$, symptoms S_ij1_ and S_ij2_ are independent. The Bayesian formula, which takes the correlation weight of the symptom vector for the posterior probability calculation into account, is presented as Equation (2):

1 $$ {\mathrm{Corr}}_{\left({S}_{ij1,}{S}_{ij2}\right)\left|{D}_f\right.}=\frac{P\left(\left({S}_{ij1,}{S}_{ij2}\right)|{D}_f\right)}{P\left({S}_{ij1}|{D}_f\right)\bullet P\left({S}_{ij2}|{D}_f\right)} $$

2 $$ \mathrm{P}\left({\mathrm{D}}_{\mathrm{f}}\left|{\mathrm{S}}_{\mathrm{i}}\right.\right)={\mathrm{Corr}}_{{\mathrm{S}}_{\mathrm{i}}\left|{\mathrm{D}}_{\mathrm{f}}\right.}\bullet \mathrm{P}\left({\mathrm{D}}_{\mathrm{f}}\right)\bullet \frac{\prod \limits_{\mathrm{j}=1}^{\mathrm{n}}\mathrm{P}\left({\mathrm{S}}_{\mathrm{i}\mathrm{j}}|{\mathrm{D}}_{\mathrm{f}}\right)}{\mathrm{P}\left({\mathrm{S}}_{\mathrm{i}}\right)} $$

For the experiment, a receiver operating characteristic curve (ROC) is utilized to evaluate the accuracy of the ontology-driven diagnosis classification in which formal measures are used to evaluate the rate of success in distinguishing the correct disease and identifying an appropriate therapeutic regimen. An ROC curve is related to the number of true positives (TP), the number of false positives (FP), the number of true negatives (TN), and the number of false negatives (FN). An ROC space is defined by the false positive rate (1 − specificity = FP ∕ (TN + FP)) and the true positive rate (sensitivity = TP ∕ (TP + FN)) as the x- and y-axes, respectively. Each prediction result produces a (1-specificity, sensitivity) pair and represents a point in the ROC space. Then, we plot the ROC point for each possible threshold value result (the threshold specifies the minimum a posteriori probability for assigning a sample to the positive class), thereby forming a curve. In this study, we use the area under the curve (AUC), whose value is typically between 0 and 1, to measure and compare the classification performances of classifiers. An AUC value of 0.5 corresponds to random predictions. A satisfactory classifier should have an AUC value that substantially exceeds 0.5. The higher the AUC value is, the better is the classification performance.

**Fig. 2:**
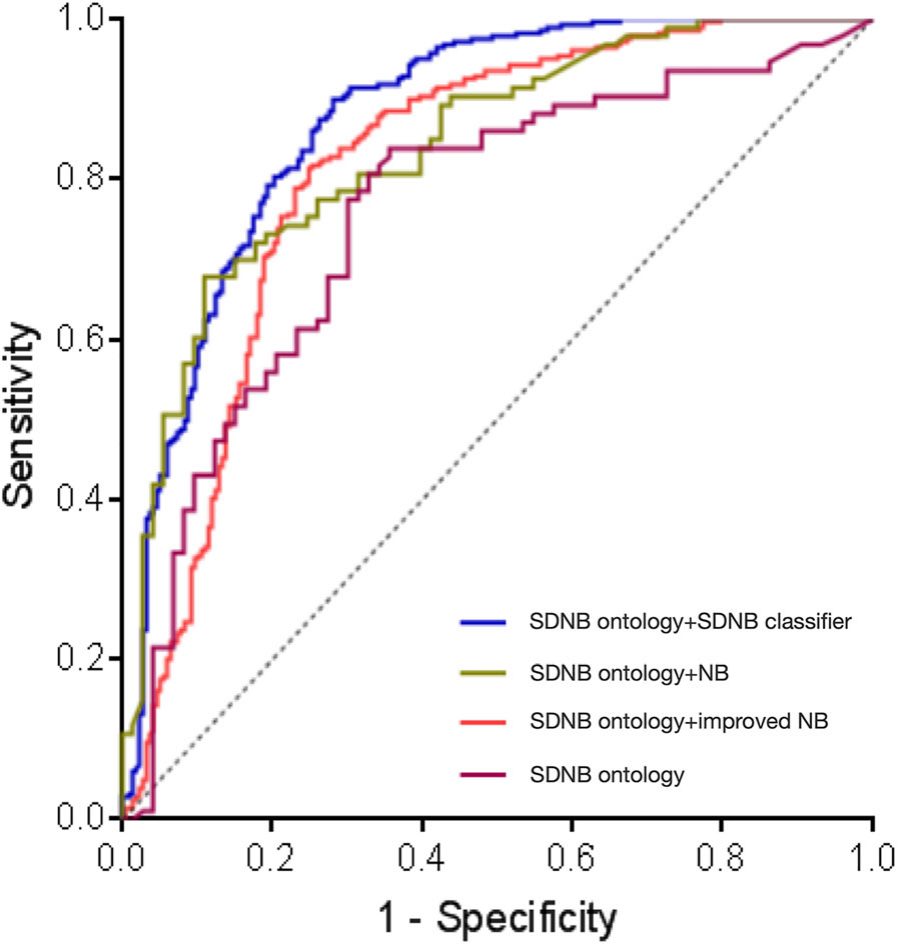
ROC chart and AUC for classifier evaluations

The ROC curves that are presented in Fig. 2 represent the simulation results. Using various threshold values, we aim at determining whether the experimental result can yield an accurate diagnosis based on various ontologies, where 0 denotes no and 1 denotes yes. The calculation of a classifier with the test data returns a probability pair, namely, [P1, P2], that specifies a probability of 0 or 1. The obtained results, such as 0: [3.63E-09, 1.00E+ 00] and 1: [0.962542578, 0.037457422], can be connected by a line and presented as ROC curves.

As shown in Fig. 2, the ROC curve that corresponds to the operation combination of the SDNB ontology and the SDNB classifier shows the highest performance at most tested noise levels, which demonstrates the effectiveness of incorporating OR value evaluation and AdaBoost optimization into the base model. The ontology that was developed with probabilities and enriched by more complete knowledge can accurately represent the relationships between diseases and symptoms and can provide superior data support for decision-making during diagnosis.

Comparing the blue curve with the red curve, the accuracy of the diagnosis has been significantly improved. This is expected since the OR value is particularly suitable for comparing the relative odds of the occurrence of disease outcomes given exposure to the health feature variable and attribute.

All ROC curves that are discussed above are obtained from the experimental results, which are listed in Table 2. The *p*-values are calculated using the GraphPad Prism 7 software based on the principle of the Z test by comparing the AUC values with 0.5. The null hypothesis, namely, H_0_, is AUC = 0.5 and the alternative hypothesis, namely, H_1_, is AUC > 0.5.

**Table 2:** Experimental results in four scenarios: (a) without the naïve Bayes classifier; (b) with the original naïve Bayes classifier; (c) with an improved naïve Bayes classifier that is based on the co-occurrence frequency; and (d) with the symptom-dependency-aware weighted naïve Bayes classifier

	SDNB ontology	SDNB ontology + NB	SDNB ontology + improved NB	SDNB ontology + SDNB classifier
Area under the ROC curve	0.7574	0.8392	0.8753	0.8876
Std. of the error	0.03865	0.03063	0.01628	0.01264
95% confidence interval	0.6817 to 0.8331	0.7792 to 0.8993	0.8434 to 0.9073	0.8437 to 0.9281
*P* value	< 0.0001	< 0.0001	< 0.0001	< 0.0001

### Diagnostic reasoning cases

Three positive sample cases that use a small part of the EMR dataset and their prediction results that are based on our generated ontology are listed in Table 3. The correctly identified diseases were the top scored diseases by each model. Our symptom-dependency-aware naïve Bayes classifier substantially and consistently outperforms the baselines, thereby demonstrating the remarkable applicability and effectiveness of our method.

**Table 3:** Diagnostic reasoning results in four scenarios: (a) without any naïve Bayes classifier; (b) with the original naïve Bayes classifier; (c) with the improved naïve Bayes classifier that is based on the co-occurrence frequency; and (d) with the symptom-dependency-aware weighted naïve Bayes classifier

Disease	Case	Symptom set	SDNB ontology	SDNB ontology + NB	SDNB ontology + improved NB	SDNB ontology + SDNB classifier
Jaundice	Case 1	{Nausea, Vomiting, Yellow sclera, Weary, Pale stools, Dark urine, Itchiness, Fatigue, Abdominal pain, Weight loss, Vomiting, Fever, Pale stools, Dark urine}	0.67	0.71	0.83	0.862
Pancreatic Cancer	Case 2	{Yellow sclera, Jaundice, Abdominal pain, Back pain, Bloating, Nausea, Vomiting}	0.54	0.61	0.64	0.646
Liver disease	Case 3	{Dizziness, Body skin yellow dyeing, Abdominal pain and swelling, Itchy skin}	0.42	0.48	0.55	0.567

**[Case 1: Jaundice]** The classification results for the four scenarios are all correct. The probability of the disease that is predicted by the symptom-dependency-aware naïve Bayes classifier is higher; hence, by taking into account the correlations among symptoms, the more symptoms the patient has, the more accurate the prediction is.

**[Case 2: Pancreatic Cancer]** The classification results for the four scenarios are correct. If there is no significant correlation among the selected symptoms, the probabilities of disease that are predicted by the baseline classifiers and the symptom-dependency-aware naïve Bayes classifier are similar.

**[Case 3: Liver disease]** The improved naïve Bayes classifier correctly classifies the disease, while the other two methods (SDNB ontology and SDNB ontology +NB) do not accurately identify the disease. For example, the predicted score for liver disease that was provided by the SDNB ontology is 0.42; hence, the total score for other possible diseases is 0.58. Scores that are not well differentiated cannot provide useful support for clinical decision-making. It is also observed that the improved naïve Bayes classifiers outperform the original classifiers if there are few symptoms but strong correlations among these symptoms.

**Fig. 3:**
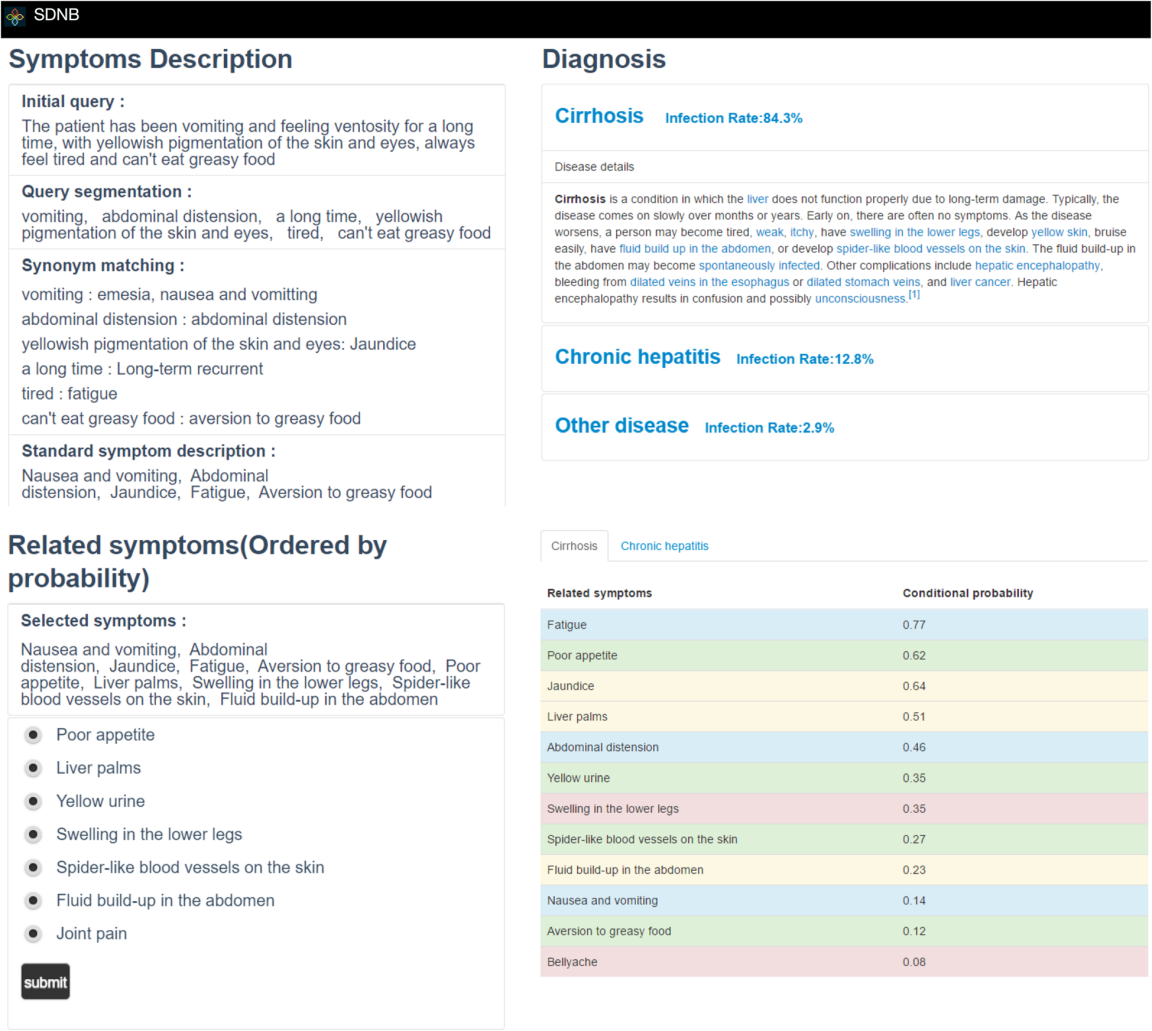
Diagnosis of cirrhosis based on the generated SDNB ontology and the proposed SDNB classifier

A typical research case that involved answering clinical queries about gastroenterological disease was developed to evaluate the diagnostic reasoning and probability computations based on the ontology (see Fig. 3). The UI interface is an HTML page that is based on the bootstrap framework.

As shown in the upper-left part of Fig. 3, after receiving an initial query from a user, our proposed model (SDNB ontology + SDNB classifier) outputs the standard symptom expressions. First, we match the input query in the SDNB ontology via ontology components “class name” and “alias” (represented by the relation “hasExactSynonym” in OWL) via n-gram text matching. Then, the detected symptoms and their synonyms are returned for the users as a reference. Finally, our model (SDNB ontology + SDNB classifier) identifies the standard symptom expressions for conducting diagnostic reasoning. Based on the involved standard symptoms, our model provides a list of relevant symptoms from which the user can select according to the entity relevance within the ontology (see the lower-left part of Fig. 3). With all selected symptoms, our model calculates the probability of illness using the proposed naïve Bayes classifier. The diagnostic results are presented in the upper-right part with a description of the possible disease. In addition, the symptoms’ conditional probabilities are presented as details in the bottom-right part and serve as references for the patient.

## Discussion

This manuscript combined research on knowledge discovery and probability discovery from EMRs with ontology completion in the medical field. This study explored a symptom-dependency-aware naïve Bayes classifier, which involves the automatic determination of probabilities between diseases and syndromes to facilitate ontology applications in probabilistic diagnosis inference.

Technically, we present a reproducible approach for learning probability information that involves diseases and symptoms from an EMR. The proposed operation depends on various methods that are based on EMRs, as described in this manuscript. In contrast to our previous approach that evaluated the attribute correlation based on the attribute co-occurrence frequency, we explore the acquisition of disease-symptom factors from EMR texts using an OR value that is especially suitable for medical applications. In our study, the OR value measures the association that compares the likelihood of disease of exposed patients to the likelihood of disease of unexposed patients. Compared with the existing ontologies, we built a more domain-specific and complete ontology for gastrointestinal diseases. The experimental results demonstrate that the direct and automated construction of a high-quality health ontology from medical records is feasible.

Practically, the proposed approach provides possible references for clinicians and ontologists. The proposed approaches can offer a quick overview of disease-relevant factors and their probability distribution to users. The learned probabilities render the ontology more interpretable.

Several limitations are encountered in this study. The disease/symptom modeling is conducted based on EMR records; thus, it is critical to have a large volume of high-quality EMR records. However, the records could easily be biased. In addition, this study focused only on the first disease that is listed in the medical record and ignored the other diseases and complications. Although this method accords with clinical logic and effectively reduces noise during the reasoning process, it will reduce the amount of useful information.

Accordingly, one of the more promising avenues for future research is the incorporation of other data-mining techniques, such as heuristic learning and clustering, for attribute distillation [41]. Meanwhile, we will study the entire diagnosis results in terms of the data integrity and distribution. A distribution plot of the numbers of identified/associated diseases per EMR record will be explored to identify important information.

## Conclusions

In this paper, we present a medical knowledge probability discovery method that is based on the analysis and extraction of EMR text data for enriching medical ontologies with probability information. The experimental results demonstrate that the proposed method can effectively identify accurate medical knowledge probability information from EMR data. In addition, we evaluate the performance of the proposed method under various scenarios, including diagnosis classification and diagnosis reasoning.

Although we have presented an application of the ontology-based Bayesian approach in gastrointestinal diseases, the search algorithm is not limited to gastrointestinal diseases. Our ontology-based Bayesian approach is amenable to a wide range of extensions that may be useful in scenarios in which the features are interrelated.

## Methods

In this section, we introduce an improved naïve Bayes classifier for triplet probability computation for conducting a medical knowledge probability discovery task and enrich the ontology with knowledge-triplet probability information.

### Ontology construction with EMRs

**Fig. 4:**
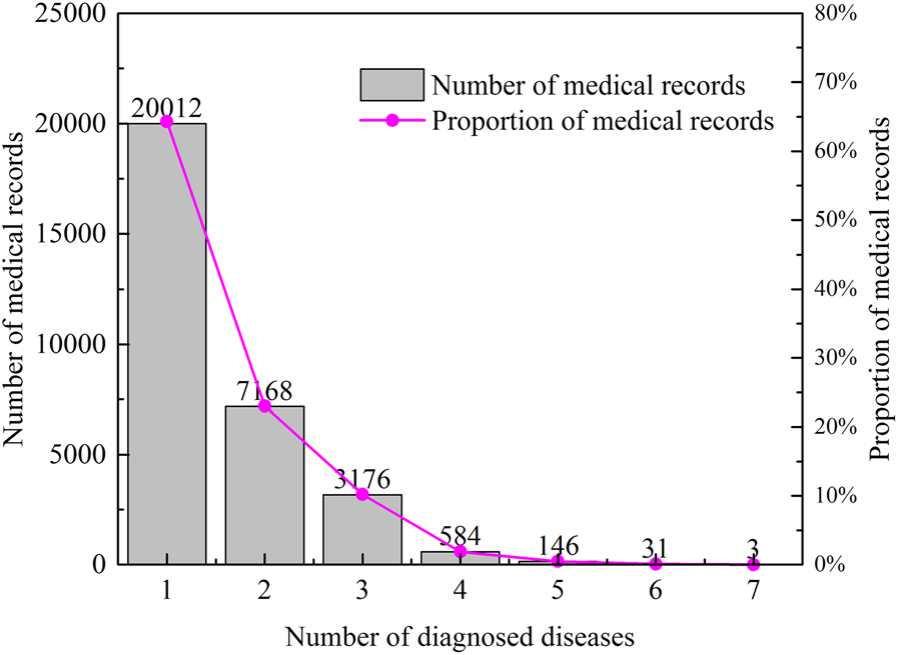
Distribution of the number of diseases diagnosed by doctors in all involved medical record data

We obtain 100,198 EMRs, collecting from February 2015 to July 2016, from a partner clinic located in a municipality of China. Among all these EMRs, 31,120 are about gastrointestinal diseases, and they are adopted as training and testing sets in this study. In the medical record, according to the patient’s symptoms, the number of diseases diagnosed by the doctor ranges from 1 to 7, and the corresponding medical records account for 64.30, 23.03, 10.21, 1.88, 0.47, 0.1 and 0.01% of the total medical records, respectively (see Fig. 4). It should be noted that we only count the primary disease listed in the medical record. For example, the EMR with ID 00292987 is about an 80 years old male, who suffers from chronic gastritis and left ureteral calculi. Since he was in the Department of Gastroenterology, the doctor focused on his primary disease chronic gastritis and listed his known long-term disease (left ureteral calculi) as other diseases.

As the EMRs are provided in the formats of image and PDF, we transform them into texts using an Optical Character Recognition (OCR) tool. At present, the accuracy of data recognition through OCR tools varies from 90 to 99% depending on the identification content. We randomly sample 20 transformed EMRs to find frequent error characters that are caused by the OCR tool. Then, based on these OCR error patterns and the EMR organization formats, we design a set of regular expressions to extract the patient fields as needed. To be more specific, the EMRs from our partner clinic can be categorized into three organization formats and have similar segmentation indicators, including “sex”, “age”, “symptom”, “diagnosis”, “admissions records”, “discharge records” and “medical history”, which facilitates the design of regular expressions.

For the proofreading of medical record data, if errors occur frequently in the same situation (e.g., when identifying information in a table, the presence of table line may result in the appearance of meaningless symbols), they would be statistically adjusted and removed. To further ensure the accuracy of text recognition, we invited three medical students to proofread all the extracted texts. According to statistics, word recognition errors that require their correction exist in less than 2% of medical records. Some common mistakes include the Chinese word “脉” being misidentified as recognized as “Sz1” for unknown reason, and the word “日” being misidentified as “曰”.

As this analysis focuses on diseases that are related to gastrointestinal diseases, we attempt to identify the medical data that pertain to gastrointestinal diseases. Based on the diagnosis results that are presented in the EMRs, we filter out those data for which the premier diagnosis is not a gastrointestinal disease. After preprocessing steps, we retain 31,720 EMR data, which correspond to different patients according to the serial numbers of the outpatient clinic and hospital.

The inputs of this task are a set of EMRs, an example of which is presented in Table 4.

**Table 4:** Example of Chinese EMR data that has been translated into English

Item	Content
GENDER	Male
AGE	48
ILLNESS_DESC	The patient complained of abdominal discomfort after meals, especially high-fat meals. He also had aching in his right shoulder and back.
BODY_EXAM	An ultrasound of the upper abdomen revealed cholelithiasis.
DIAG_DESC	Cholecystitis

The EMR texts are in Chinese and require word segmentation to divide the text into Chinese component words. In this paper, we use a Chinese word segmentation tool, namely, jieba,^1^ to generate the tokenized causal-mention sentences.

We use the International Classification of Diseases (ICD-10) in the Chinese language and the largest medical e-dictionary^2^ for word matching. The e-dictionary contains 12 million terms in Chinese, which cover vocabulary in various clinical departments, basic medicine, molecular biology, medicines, instruments and traditional Chinese medicine. Selecting these two medical dictionaries as the target, we perform n-gram entity name matching to extract medical entities from raw texts. Typically, an n-gram is a contiguous sequence of n items from a specified sample of text.

The disease-symptom mentions are extensive in EMR data. The patient usually describes his/her symptoms and medical history with explicit temporal and causal indicators (e.g., “before”, “after”, and “since”), while the doctor usually provides diagnosis and therapy suggestions in response to questions, in which the doctor refers to symptoms and diseases, along with their relationships. The mentions of lesions, pathologies, and susceptible populations, among others, are also extracted. Then, we match entity pairs in the same text to possible knowledge triplets using an alias table. Via this approach, we extract the knowledge triplets from the raw medical data.

**Fig. 5:**
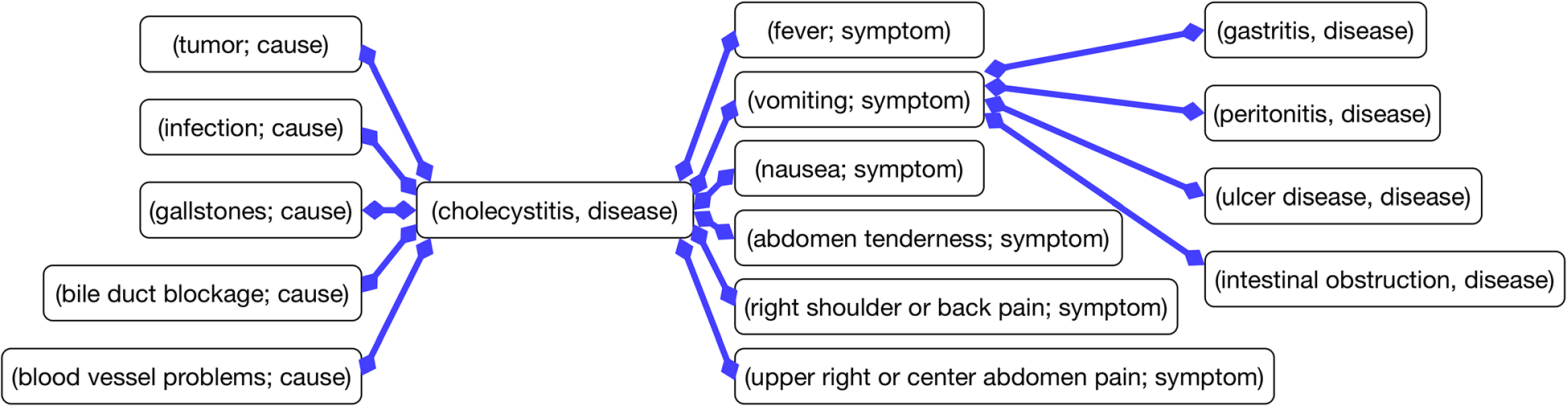
Subgraph of the generated ontology

Afterwards, we add the entity tag in the EMR data to each matched entity and the triplet is transformed into an entity pair: (entity1; tag1) → (entity2; tag2) (e.g., (catch-a-cold; symptom) → (fever; disease)). The same entity may have multiple tags (e.g., a disease can become a symptom under various clinical conditions) and play multiple roles in the ontology. Finally, such triplets are composed as an ontology by combining the aliases (see Fig. 5).

Via entity name matching, the knowledge of gastrointestinal system diseases^3^ in the disease ontology is adopted to enrich the generated ontology. Consider the disease “allergic bronchopulmonary aspergillosis” as an example. We can obtain its superclass (aspergillosis), disease ID (DOID:13166) and other cross-reference information (e.g., OMIM:103920, MESH:D001229, and ICD9CM:518.6).

However, the generated SDNB ontology is not sufficiently accurate for use because there is no information that explicitly specifies the probability of the co-occurrence of a disease and a symptom. In the remainder of this section, we introduce an improved naïve Bayes classifier for conducting probability discovery.

### Symptom-dependency-aware Naïve Bayes classifier

We propose a symptom-dependency-aware naïve Bayes classifier that is based on the assumption that symptoms have a level of dependency among them. The proposed naïve Bayes classifier calculates the probability that a patient is suffering from a specified disease and outputs the relevant symptoms of that disease. Afterwards, via innovative approaches, we incorporate the value of the probability of a disease into the ontology.

**Fig. 6:**
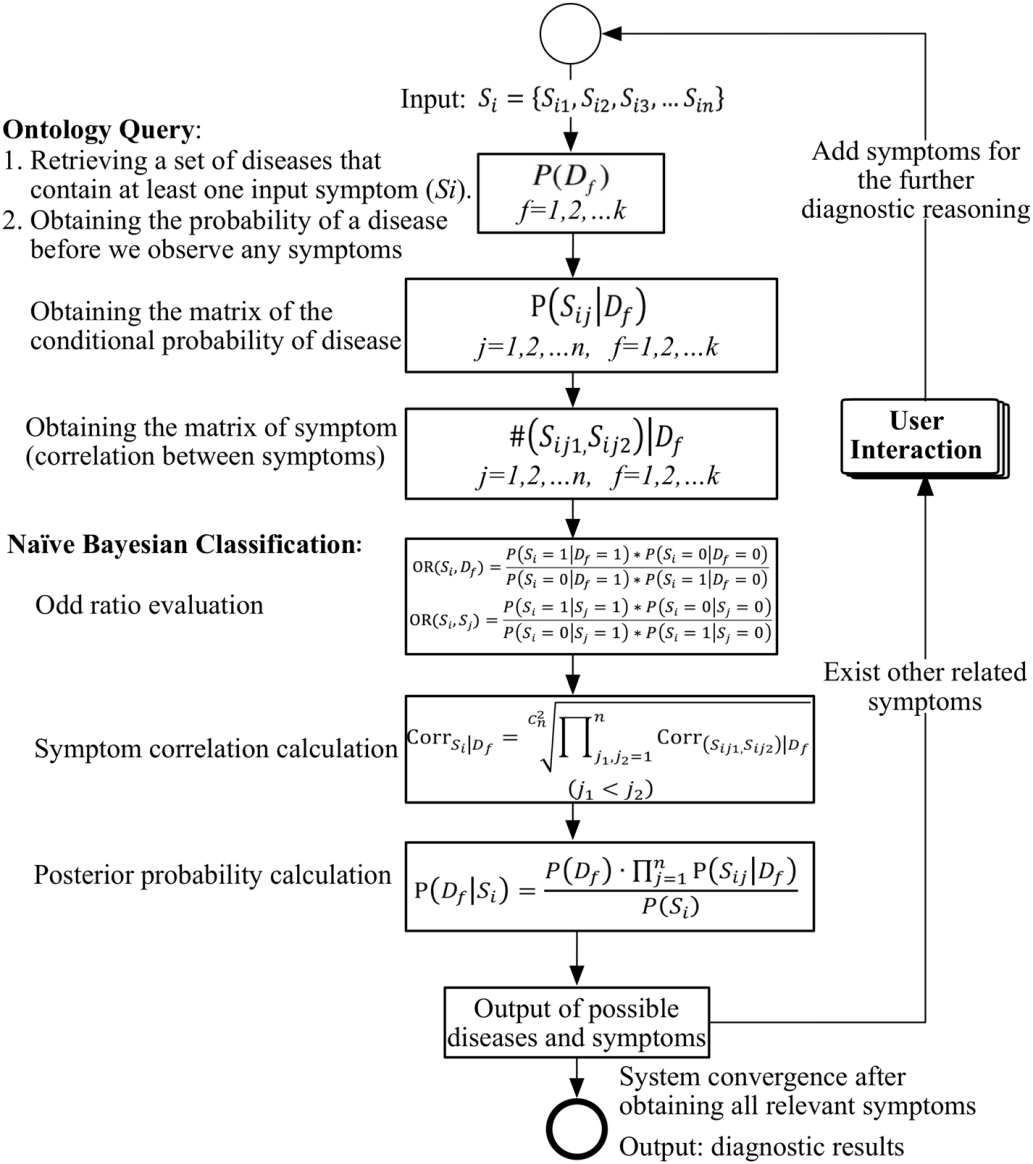
Flow diagram of disease probability calculation using the improved naïve Bayes classifier based on attribute relevance

Figure 6 shows a flow diagram for calculating the disease probability using the symptom-dependency-aware naïve Bayes classifier. The calculation process includes ontology queries and naïve Bayes classification. During the gastroenterology diagnosis, the proposed method reads the proposed ontology using Java code to query the following information in the ontology: a disease and its relevant symptoms, the probability of a disease before we observe any symptoms, and the conditional probability of a symptom given a disease. All this information is considered as the basis for classification.

Then, the naïve Bayes classification steps determine the probabilities that various diseases will occur when symptom *S*_*i*_ occurs. Finally, the classifier outputs a set of diseases that have high probabilities and other symptoms that are associated with these diseases. Our model allows the user to select additional relevant symptoms as a supplement to the initial query. The classifier will continue to operate until the user completes symptom selection, at which point the diagnosis results will be complete.

#### Naïve Bayes

Formally, we consider *k* disease categories, namely, {*D*_1_, *D*_2_, *D*_3_ … *D*_*k*_}, and *m* diagnostic samples, namely, {*S*_1_, *S*_2_, *S*_3_, …*S*_*m*_}, where each sample contains *n* symptom attributes, which are denoted as *S*_*i*_ = {*S*_*i*1_, *S*_*i*2_, *S*_*i*3_, …*S*_*in*_}.

Equation (3) expresses the naïve Bayes computation, where *P*(*D*_*f*_) denotes the probability of disease *D*_*f*_ before we observe any symptoms. We obtain *P*(*D*_*f*_) based on statistical results or expert experiences. Given a symptom *S*_*i*_, P(*D*_*f*_| *S*_*i*_) is the posterior probability of *D*_*f*_.

The conditional probability of *S*_*i*_ equals *P*(*S*_*i*_| *D*_*f*_) if D_f_ holds. Here, $\frac{P\left({S}_i|{D}_f\right)}{P\left({S}_i\right)}$ can be treated as an adjustment factor for the disease probability P(D_f_). If the adjustment factor is > 1, *P*(*D*_*f*_) will be augmented; hence, the probability of occurrence of disease D_f_ is higher; if the adjustment factor is < 1, *P*(*D*_*f*_) will be weakened; hence, the probability of occurrence of *D*_*f*_ is lower. If the value of the adjustment factor = 1, the probability of occurrence of disease *D*_*f*_ is unaffected.

3 $$ \mathrm{P}\left({D}_f|{S}_i\right)=\frac{P\left({D}_f\right)\bullet \mathrm{P}\left({S}_i|{D}_f\right)}{P\left({S}_i\right)} $$

According to the assumption of attribute independence, which underlies naïve Bayes, the Bayesian multiplicative equation can be simplified to Equation (4):

4 $$ \mathrm{P}\left({D}_f|{S}_i\right)=\frac{P\left({D}_f\right)\bullet \prod \limits_{j=1}^n\mathrm{P}\left({S}_{ij}|{D}_f\right)}{P\left({S}_i\right)} $$

#### Symptom-dependency-aware Naïve Bayes classifier

A symptom-dependency-aware naïve Bayes classifier is designed based on the attribute relevance. Naïve Bayes evaluates the correlation between symptoms in terms of the dependency degree between symptom vectors. The conditional probability of a symptom vector is evaluated as the product of the conditional probability of each symptom and the dependency degree of the symptom vector. By calculating the symptom vectors, the probability of a disease, namely, *P*(*D*_*f*_), is used to estimate its posterior probability.

1. Correlations between symptoms

As expressed in Equation (5), the OR value between any two nodes is evaluated based on the co-occurrence frequency among symptoms in the EMRs. Using 30,060 EMR data as training set, a threshold of at least 5 co-occurrences between symptom pairs was selected as a denoising measure. Here, 5 corresponds to the number of co-occurrences between symptom pairs in each EMR record. We experimented with several co-occurrence thresholds (0, 2, 5 and 10) and selected the smallest value that performed well in the automatic evaluation. According to the pre-experiment, the number of EMRs has little impact on the threshold setting.

The OR value can be used to estimate the mutual information strength between symptom S_i_ and disease D_f_. If the OR between symptom *S*_*i*_ and disease *D*_*f*_ exceeds 1, then having symptom *S*_*i*_ is considered to be a risk factor for disease *D*_*f*_. If the OR value is less than 1, symptom *S*_*i*_ is not highly relevant to disease *D*_*f*_:

5 $$ \mathrm{OR}\left({S}_i,{D}_f\right)=\frac{P\left({S}_i=1|{D}_f=1\right)\ast P\left({S}_i=0|{D}_f=0\right)}{P\left({S}_i=0|{D}_f=1\right)\ast P\left({S}_i=1|{D}_f=0\right)} $$

To estimate the mutual information between symptoms, namely, to quantify how strongly the presence or absence of symptom *S*_*i*_ is associated with the presence or absence of symptom *S*_*j*_, we simultaneously calculate OR(*S*_*i*_, *S*_*j*_) as:

6 $$ \mathrm{OR}\left({S}_i,{S}_j\right)=\frac{P\left({S}_i=1|{S}_j=1\right)\ast P\left({S}_i=0|{S}_j=0\right)}{P\left({S}_i=0|{S}_j=1\right)\ast P\left({S}_i=1|{S}_j=0\right)} $$

Based on the obtained OR value, the correlations between the symptoms is:

7 $$ {\mathrm{Corr}}_{\left({S}_i,{S}_j\right)\left|{D}_f\right.}=\frac{\mathrm{OR}\left({S}_i,{S}_j\right)}{\mathrm{OR}\left({S}_i,{D}_f\right)\bullet \mathrm{OR}\left({S}_j,{D}_f\right)},\left(j!=i\right) $$

2) The symptom-dependency-aware naïve Bayes classifier that is based on attribute relevance

The improved formula, which evaluates the posterior probability by taking into account the dependency degree of the symptom vector, is presented as Equation (8):

8 $$ \mathrm{P}\left({D}_f|{S}_i\right)=\frac{{\mathrm{Corr}}_{S_i\left|{D}_f\right.}\bullet P\left({D}_f\right)\bullet \prod \limits_{j=1}^n\mathrm{P}\left({S}_{ij}|{D}_f\right)}{P\left({S}_i\right)} $$

where ${\mathrm{Corr}}_{S_i\left|{D}_f\right.}$ denotes the dependency degree of symptom vector *S*_*i*_, which can be calculated via Equation (9). There are *n* symptoms and ${C}_n^2$ denotes the number of pairwise symptom combinations:

9 $$ {\mathrm{Corr}}_{S_i\left|{D}_f\right.}=\sqrt[{C}_n^2]{\prod_{i,j=1}^n{\mathrm{Corr}}_{\left({S}_i,{S}_j\right)\left|{D}_f\right.}}\ \left(j<i\right) $$

The main strategy is to represent the dependency degree of a symptom vector as the correlation product of symptom pairs approximately, since the dependency degree of the symptom vector is proportional to the correlations between the pairs of symptoms.

3) Optimization of the Symptom-dependency-aware Naïve Bayes classifier

Adaptive boosting (AdaBoost) [42] is used to optimize the proposed naive Bayes classifier. AdaBoost randomly selects the symptom vectors from the training database and trains the proposed classifier on the selected subset. The remaining data are used as test data. Vectors that are misclassified will form the subset for training; hence, the proposed classifier will learn the misclassified symptom vectors in the next round.

We utilize the effect of the number of symptoms in the symptom vector to smooth the product by calculating the correlation coefficient. The training process is described as follows:

- [Step 1] Sample Statistics.

We count the number of samples #*D*_*f*_ for disease *D*_*f*_, the number of samples #*S*_*ij*_|*D*_*f*_ in which symptom *S*_*ij*_ is associated with disease *D*_*f*_, and the number of samples #(*S*_*i*,_*S*_*j*_)|*D*_*f*_ in which symptom pair (*S*_*i*,_*S*_*j*_) occurs with disease *D*_*f*_.

- [Step 2] Disease and Symptom Probability Evaluation.

Using the results from the sample statistics, the probability of a disease, namely, *P*(*D*_*f*_), and the conditional probability of a symptom, namely, *P*(*S*_*ij*_| *D*_*f*_), can be calculated via Equation (10) and Equation (11), respectively:

10 $$ P\left({D}_f\right)=\left({\mathrm{Count}}_{{\mathrm{D}}_{\mathrm{f}}}+1\right)/\left(\mathrm{m}+\mathrm{k}\right) $$

11 $$ P\left({S}_{ij}|{D}_f\right)=\left({Count}_{S_{ij}\left|{D}_f\right.}+1\right)/\left({Count}_{D_f}+k\right) $$

where *m* is the number of samples in the training set *S* and *k* is the number of diseases. The Laplace correction (the “+ 1” in the numerator and the “+ k” in the denominator) is utilized to estimate probabilities in machine learning.

- [Step 3] Pairwise Symptom Conditional Probability and Symptom Correlation Matrix.

We estimate the conditional probability P((*S*_*i*,_*S*_*j*_)|*D*_*f*_) of symptom pair (*S*_*i*,_*S*_*j*_). The correlation of each symptom pair is evaluated via Equation (7) to produce a matrix of symptom correlations.

In the classification process, given the symptom vectors, we calculate the posterior probability of a disease and select the disease that has the maximum posteriori probability.

- [Step 1] Vector Correlation.

Given a test sample *S*_*i*_ = {*S*_*i*1_, *S*_*i*2_, *S*_*i*3_, …*S*_*in*_}, the dependency degree ${Corr}_{S_i\left|{D}_f\right.}$ of symptom vector *S*_*i*_ is calculated via Equation (9) with the symptom correlation matrix.

- [Step 2] Symptom Posterior Probability and Diagnosis Classification.

We calculate the disease posterior probability *P*(*D*_*f*_|*S*_*i*_) via Equation (8) and select the diseases with high posteriori probability values as the diagnosis classification results.

### Enriching the ontology with probabilities

After obtaining the disease- and symptom-relevant probabilities via the symptom-dependency-aware naïve Bayes calculation, we need to add the values of the probabilities into the ontology.

A MySQL database is used to store the disease probability and symptom conditional probability that were evaluated via the original naïve Bayes classifier or the improved naïve Bayes classifier. The data conversion between this MySQL database and the ontology in web ontology language (OWL) is conducted by the Owlready package [43]. The probability values of a disease are added to DataProperty of the ontology rather than to AnnotationProperty. Thus, the ontology metrics can be calculated by Protégé and read by Owlready, rdflib or any other ontology development tool [44]. Via this approach, the symptom-dependency-aware naïve Bayes classifier can perform the disease probability calculation.

## Data Availability

Source code about the symptom dependency-aware Naïve Bayes probability computation and the ontology are accessible via: https://github.com/shenyingpku/IASO

## Abbreviations

AUC: Area under the Curve
DO: Disease Ontology
EMRs: Electronic medical records
FN: Number of false negative
FP: Number of False Positives
GIDEON: Global Infectious Disease and Epidemiology Network
OWL: Web ontology language
PRA: Path ranking algorithm
ROC: Receiver operating characteristic curve
SDNB: The name of the proposed classifier and the generated ontology
TN: Number of true negatives
TP: Number of true positives

## Footnotes

1
https://github.com/fxsjy/jieba

2
http://dic.medlive.cn

3
https://www.ebi.ac.uk/ols/ontologies/doid/terms?iri=http%3A%2F%2Fpurl.obolibrary.org%2Fobo%2FDOID_77
